# Is Dentistry a Profession?

**Published:** 1886-10

**Authors:** W. C. Wardlaw

**Affiliations:** President Southern Dental Association


### Is Dentistry a Profession?

ANNUAL ADDRESS BY DR. W. C. WARDLAW,

President Southern Dental Association, July 28, 1886.

*Gentlemen of the Southern Dental Association:*

In discharging this constitutional duty which your kindness has devolved upon me, I desire to address you a few thoughts upon the relationship of dentistry to the science of medicine.

Is dentistry a distinct profession, or should it be regarded as a specialty of medicine? In royal old England, so conservative of custom and so zealous of dignity, the latter claim would not be allowed, but in democratic America a more liberal sentiment obtains, and such a relation is gradually being accorded.

In England to-day, the "College of Physicians," those mighty men of powdered wig and gold-headed canes, frowns down "specialism," even in surgery and the business of the apothecary, and their conservative instincts still protest against the "specialist." The battle is even now waging there, with victory smiling on "specialism." As it is conceded that America has been the leader of England in most matters pertaining to dentistry, we may hope that in this instance, too, she may succeed in breaking down some of these conventional barriers standing in the way of progress. Our claim is that, while dentistry is a distinctly organized profession, made so by peculiar circumstances, it is properly and really a specialty of medicine, being at the same time a science as well as an art, and should be so recognized and encouraged. The medical fraternity generally are disposed to regard it as one of the arts, with a slight admixture of medicine.

Medicine, in the present acceptation of the term is very comprehensive, and signifies all that pertains to the prevention, alleviation, and cure of disease, being conveniently divided into surgery and medicine. The ancients did not recognize a distinction between these two, but included both in the "Healing Art." We, however, mark the essential points of difference by limiting surgery to the concern of local injuries and disorders, and restricting medicine to those affections which involve the general system.

Homer does in his "Siege of Troy" allude to the two sons of Æsculapius as being the one, a skillful surgeon, and the other a wise physician, but that this division was very marked or long continued, history does not inform us.

General surgery is arbitrarily sub-divided, according to the particular part or organs of the body treated, into ophthalimic surgery, obstetric surgery, laryngal surgery, oral surgery, etc. Dentistry, being almost synonymous with oral surgery, would thus seem to be a part, and a special part of medicine. But not having been so taught by medical colleges, and rather repudiated by them, how has it been evolved as a specialty of medicine?

The vast and ever increasing domain of medicine would seem to preclude the possibility of the whole of its being properly cultivated by one mind.

Necessity and convenience have therefore divided and subdivided it into various branches and departments. Long before the accruing knowledge of modern times embraced this necessity, the ancients had found it expedient to do the same thing. Herodotus tells us that in Egypt the mother of the arts and sciences, "so wisely was medicine managed, there were physicians, each of whom applied himself to one disease, and only one; some are for the eyes; others for the head; others for the teeth, and others internal diseases." This system, however, did not prevail universally, and, during the slow advance of many years, the individual practitioner was expected to combine in his one person all attainable knowledge of the "healing art."

But, as each upward step upon the mountain side widens the range and extends the view, successively bringing to sight new and varied beauties of nature, until the farthest reach of vision is unable to take it all in, so the onward march of later study and research in medicine has led to the discovery of new truths and the development of new principles, until now no one intellect is of such giant proportions as to be able to grasp them all. Necessity, I say, has therefore decreed that it should be parceled out amongst a number of colaborers, each pursuing that branch recommending itself to his preference, and so, one and another becoming skillful and proficient in his special branch, would naturally be acknowledged as an "expert," the modern "specialist."

This was perhaps first observed in the disposition of some physicians to limit their practice to that of surgery proper.

Division of labor, mental and manual, tends to proficiency of attainment. We may ridicule the man with a "hobby," as the one-ideaed man, but it takes the man with one idea, persistently and energetically following it to carry it to a successful issue. A Jack-at-all-trades is very apt to be good at none. He is most apt to excel in art or science, who in the choice of a life vocation is wise enough to select that one most congenial to his taste and in accordance with the bent of his genius. Having so chosen, and through application and ambition made it a success, it is his mission to pursue it, because therein he can do the most good for his fellow-man.

The modern "specialist" is, therefore, a desirable and legitimate personage. Accordingly we have in medicine the "specialist" of the eye, the ear, the thoracic cavity, the urinary organs, of nervous disorder, of skin diseases, uterine affections, etc., and why not the "specialist" of the mouth?

This status of affairs, however, has only been brought about after a long and bitter contest. Only recently in America the great Marion Sims was bold enough in courage and sufficiently independent in fortune and reputation to be able to break away from the trammels of arbitrary custom and successfully advocate the professional recognition of "specialists"

In comparatively late years it was against the "code of ethics" of the profession—'twas quackery—'twas undignified, unbecoming, for a regular practitioner to claim, however modestly, special merit or superior skill in any particular department to which he may have given attentive study.

This prejudice still obtains to a considerable extent, and seems to attach especially to dentistry—why, I am at a loss to understand, unless it be that in its practice it has much of art, and the mechanical arts do not recommend themselves to that pride of intellect which is accustomed to look with contempt upon manual labor, as not altogether respectable. General surgery is art, high art, utilizing numerous mechanical instruments and appliances, and requiring dexterous skill, an educated eye, and a just perception of the beautiful.

These are essential elements of dentistry, too, and it should not be accorded any less respect and honor on account thereof.

The cases are similar thus far, but the difference which seems to make the distinction is that dentistry has not been acquired in a medical school as surgery has been. Dentistry, as a matter of fact, is not covered by a medical diploma, and to that extent cannot technically be a "specialty" of medicine. What is a diploma, however, but a mere certificate of proficiency? If bestowed on one lacking the requisite qualifications it no more makes him an M.D. than does the withholding of it from a worthy man make him any the less a doctor in reality.

Dentistry, from the very nature of things, has grown up outside of medicine. 'Tis like the deserted child, who, disowned by its mother and brought up on the bottle, survives in spite of its hard usage. Medicine had already stepped forth in the pride of well developed proportions, when dentistry began to take its infantile steps, in its practice as a rude art by the uneducated barber. But, as one by one, men of observation would follow out its practical teachings to their logical results, 'twas found that the fundamental principles of medicine underlay them all.

These convictions gradually enforced themselves, until the ambitious artist felt impelled to closely study those branches of medicine which he recognized as bearing most intimately upon his own calling.

He studied medicine in dentistry because he could not study dentistry in medicine. The wants of her unrecognized child had not been foreseen and provided for by the unnatural mother, and only as circumstances allowed, could they be supplied by itself. The upward progress was toilsome and tedious. Dark ignorance had to be enlightened. Contracted selfishness had to be overcome, blind prejudices had to be dispelled, the sunlight of truth and science had to be admitted. All of this had to be accomplished without the encouragement, and against the opposition of the medical fraternity. Pride, prejudice, and short-sightedness occupied the place of intelligence, liberality, and penetration. Our revered friend, Dr. Thackston, doubtless remembers of his own personal knowledge and experience how Dr. Chapin A. Harris and his coadjutors made overtures to the medical colleges, endeavoring to have dental professorships incorporated in them; how their propositions were scornfully rejected; how they were driven to the necessity of establishing an independent school, and how the Baltimore Dental College, the first college of dentistry ever founded, had to struggle and strive for an early existence. And thus it was dentistry came to be taught in separate schools, and to be independent of medical colleges. The dental schools, however, are mere duplicates of the medical schools in anatomy, physiology, pathology, chemistry, materia medica, etc., and the dental students might study these branches as well in one as the other.

As time and research extends the spheres of both professions, the necessary provision will probably be made for M.Ds. and dentists being taught and graduated by the same corps of teachers. Harvard University has the honor of having led off in this direction, and has been, and will be followed by other universities. My own judgment leads me to think that dentistry pure will be better taught in dental colleges.

Year by year, as our standard of qualification is being raised, men of character and education are joining our ranks, and are being recognized by physicians as professional equals, being called into consultation with them as occasion requires.

Let us each, therefore, feel resting upon himself individually the responsibility to uphold the honor and maintain the dignity of our loved profession and there will be no need of cringing and fawning to secure that honorable recognition of which we are worthy.

I wish here to say, that in my estimation, no one instrumentality exerts more potent influence in promoting the dignity of our profession than these association gatherings.

Associative effort seems to be the characteristic feature of this day of progress. Everywhere and on all sides we have associations and conventions — agricultural, commercial, scientific, literary, and religious

Man is a gregarious animal. No one lives to himself. We cannot stand alone. We are mutually dependent. Independence is a myth. Even our every word is learned from, and belongs to, some one else.

Originality is a scarce article. All of our ideas are evolved from those of others. We may pride ourselves upon the conceptions of our fertile brains, but a strict analysis of our mental processes would reveal to us the thoughts of other minds disguised in the habiliments of our own words. There must be a reciprocity of ideas, methods, and experiences and here we have it.

As natural fruits of our Association, I mention laws regulating the practice of dentistry, State Examining Boards, the National Association of Dental Examining Boards, and the National Association of Faculties of Dental Colleges, all exercising a reciprocal influence upon education, that foundation stone upon which our professional edifice should stand. The laws gave us our Examining and Licensing Boards. As their usefulness increased, and their functions enlarged, the State Boards realized the importance of co-operation and correspondence of action, and hence was evolved the "Association of Examining Boards." These Boards, National and State, exerted such marked influence upon the colleges, as to cause them to advance their standards and league together in their "Association of Faculties."

This is the direction in which this good work should go on. Let our Boards year by year systemize their plans and perfect their workings until they are in entire accord and harmony with each other, and until a license issued in one State will be of binding force in all the States, and the diplomas of all the colleges will be received with respect and authority wherever presented.

Having reached this point we will be prepared to take the grand culminating step which will place us clearly in the lead of the medical profession. I mean, and this is the point, if point it has, of my address, that the dental profession should confer upon its worthy students a national degree which should subordinate or abolish the various degrees of D.D.S., D.M. D., L.D.D., etc., and be analagous in rank, reputation and dignity to the great English degree, "Fellow of the Royal College of Physicians." This is the great desideratum to which we must direct our efforts, but of which I haven't time to say more now. It may be unknown to some here, that we are even now leading the medical fraternity in matters of law and education. Almost nothing is being done by it in these directions.

It was my good fortune recently to attend a meeting of a State Medical Association where this was clearly manifested; there were no papers, and no discussions on education. I was surprised, and mortified to learn the very low average grade of scholarship of medical students generally, and the comparative ease with which a medical diploma may be obtained.

Their State laws are very imperfect, affording no reciprocal protection. They have no examining boards, and nothing like an Association of Faculties. Any bogus diploma will pass muster, and any ignoramus or brass-cheeked pretender may legitimately go forth to practice destruction.

In this great State of Georgia there seems to be no legal method to arrest the depredations of impostors and mountebanks.

At the very time the Georgia State Dental Society was successfully prosecuting in the City Court of Augusta, violators of the law against illegal practice of dentistry, a committee of physicians was running around consulting lawyers, hunting offices and devising measures in the futile efforts to prevent an oily-tongued, jewel-bedecked and brass mounted quack doctor from maltreating the citizens, and reaping a rich harvest of ducats from the hard earnings of his deluded victims.

They were really powerless to do more than grit their teeth and look on with smothered curses.

But I merely touch on these educational matters, knowing that we are to enjoy a rich intellectual repast in some of the papers to be offered on them.

Other matters looking to our higher advancement, and suggesting themselves for consideration and action, might be mentioned—the appointment of dentists to the army and navy, and on the National Board of Health, our share in congressional appropriations for scientific research, the approaching International Medical Congress, etc., but I have done.

Gentlemen, has not dentistry, with her many inventions, her valuable discoveries, and her signal triumphs, fairly earned for herself a niche amongst the sciences, and has she not before her a future of glorious possibilities?

Only two Sundays ago I heard for the first time in my life, specific and honorable mention made in a public address, of dentistry as a distinct and independent profession. An eloquent minister of the gospel in glowing and complimentary terms referred to it as a new science, lately evolved, which had taken exalted position among the highest and most important professions of this age. I thank you for your courteous attention.

